# Solar Doppler Velocity Results from the SDO EVE Level 4 Lines Data Product

**DOI:** 10.1007/s11207-026-02727-w

**Published:** 2026-09-09

**Authors:** Thomas N. Woods, Phillip C. Chamberlin, Dave Crotser, Andrew Jones, Rita Borelli, Don Woodraska, Gabi Gonzalez

**Affiliations:** Laboratory for Atmospheric and Space Physics, University of Colorado, Boulder, CO USA

## Abstract

NASA’s Solar Dynamics Observatory (SDO) Extreme-ultraviolet Variability Experiment (EVE) observes solar full-disk extreme ultraviolet (EUV) spectral measurements over the spectral range of 6 nm to 106 nm with 0.1 nm spectral resolution. A new EVE data product called the EVE Level 4 Lines data product provides line profile-fit results for intensity, wavelength shift, and line width for 70 emission features. These emission features are from the chromosphere, transition region, and corona. While EVE’s Multiple EUV Grating Spectrographs (MEGS) were not specifically designed to measure intrinsic solar wavelength shifts (Doppler velocities), the high stability of solar pointing by the SDO spacecraft and the very stable thermal environment for SDO in a geosynchronous Earth orbit (GEO) enable detection of solar flare Doppler velocities with MEGS spectra at its modest 0.1-nm resolution. These Doppler velocity results are more accurate after making corrections for MEGS optical wavelength shifts based on wavelength and flare location relative to the MEGS optical center. These Doppler velocity results are most interesting for studying the plasma dynamical behavior during a flare’s impulsive phase and gradual phase. In particular for eruptive flares, there are often wavelength red shifts (downflows) for the transition region emissions with Doppler velocities of about 30 km/s and wavelength blue shifts (upflows) for the corona emissions with Doppler velocities of about − 80 km/s. The Doppler velocity maxima during flare events almost always occur during the impulsive phase of the flares.

## Introduction

The Extreme-ultraviolet Variability Experiment (EVE) on NASA’s Solar Dynamics Observatory (SDO) was developed to study the solar extreme ultraviolet (EUV: 10 – 120 nm) and soft X-ray (SXR: 0.1 – 10 nm) spectral irradiance magnitude and its variability to help improve the understanding on how and why the solar EUV irradiance varies and on its impacts in Earth’s ionosphere and thermosphere (Woods et al. 2012). The emergence and evolution of the active regions drive the variability of most EUV and SXR emissions from hours to months and over the 11-year solar activity cycle, and the hot (> 6 MK) corona emissions are most energetic during flares (e.g., Lean et al. 2011; Chamberlin, Woods, and Eparvier 2008; Chamberlin et al. 2020). The solar EUV and SXR radiation is the primary energy input to Earth’s upper atmosphere to create the ionosphere and heat the thermosphere (e.g., Huba, Joyce, and Fedder 2005; Solomon, Qian, and Woods 2013), as well as a myriad of space weather impacts, such as affecting satellite drag and being disruptive for some of our navigation and communication systems (e.g., Schrijver and Siscoe 2010). Details about the EVE instruments’ designs and calibrations are provided by Woods et al. (2012), Hock et al. (2012), Didkovsky et al. (2012), and Woods et al. (2026). This paper’s primary goals are to introduce the new EVE Level 4 Lines data product and to provide some solar flare Doppler velocity results using that data product. This paper is considered a companion paper to the EVE review paper about flare observations during the SDO mission (Woods et al. 2026), and this paper provides additional results about Doppler velocities during solar flares.

The new EVE Level 4 Lines data product provides line profile-fit results for intensity, wavelength shift, and line width for 70 emission features in the 6 nm to 106 nm range. These emission features are from the chromosphere (4500 K to 25,000 K), transition region (25,000 K to 0.6 MK), and corona (> 0.6 MK), with those nominal temperature ranges adopted from Wilhelm et al. (2007). While EVE’s Multiple EUV Grating Spectrographs (MEGS) were not specifically designed to measure intrinsic solar wavelength shifts (Doppler velocities), the ability to measure small wavelength shifts in MEGS spectra has been enabled by the high stability of solar pointing by the SDO spacecraft and the very stable thermal environment for SDO in a geosynchronous Earth orbit (GEO). This Level 4 Lines (L4L) data product is described in Section 2, and the wavelength shifts due to the MEGS optical design are discussed in Section 3. Solar flare Doppler velocities, as discussed in Section 4, are observable using the L4L product after making corrections for the MEGS optical wavelength shifts.

MEGS solar irradiance measurements, being full-disk observations, cannot provide spatial information about the flare events, but can provide “flare spectra” by the simple technique of subtracting the pre-flare spectra from the irradiance spectra during the flare event. These flare spectra are most insightful into flare physics when there is just one main active flare, which is true most of the time. Solar scientists have been using solar SXR irradiance measurements to study solar flares for more than 50 years using the GOES X-Ray Sensor (XRS) measurements aboard the NOAA GOES satellites. These GOES XRS measurements provide not only space weather operational alerts for flare events but also an indication of corona temperature and emission measure changes during flares (e.g., Garcia 2000; White, Thomas, and Schwartz 2005; Woods et al. 2025). The EVE EUV Spectral Photometers (ESP) channel also provides similar SXR irradiance measurement in the 1 – 7 nm bandpass, and those measurements are also useful for nowcasting flare events.

Hudson et al. (2011) showed that wavelength shifts for some of the EVE emission features are sensitive enough to detect the SDO orbital Doppler velocity of ± 3 km/s. Thus there was promise that EVE wavelength shift analysis might also yield information about active region Doppler velocities (noting that solar surface Doppler velocities are in the ± 2 km/s range). Hudson et al. (2022) and Fitzpatrick and Hudson (2022) showed surprising results of about 50 km/s for prograde-rotation Doppler velocities with several of the EVE emission features and with the corona rotation Doppler velocities being much larger than the transition region rotation Doppler velocities. As shown later in Section 3, the EVE wavelength shifts related to the active region location are manifested primarily by the MEGS optical design being sensitive to active region location. Consequently, these prograde-rotation Doppler velocities results can be dismissed from being solar related but instead are related to the MEGS optical design being sensitive to active region location.

While EVE’s wavelength shifts from solar rotation are dominated by MEGS optical design behavior, there are valid MEGS Doppler velocity measurements during flares if MEGS spectra are properly corrected. Several have shown useful Doppler velocities results with EVE spectra during flare events (e.g., Hudson et al. 2011; Brown, Fletcher, and Labrosse 2016; Otsu and Asai 2024; Woods et al. 2026), and those results motivated the development of the EVE Level 4 Lines data product. As discussed in more detail in Section 4, this new data product is available to more easily evaluate Doppler velocity results during flare events.

## EVE Level 4 Lines Data Product

**Figure 1:**
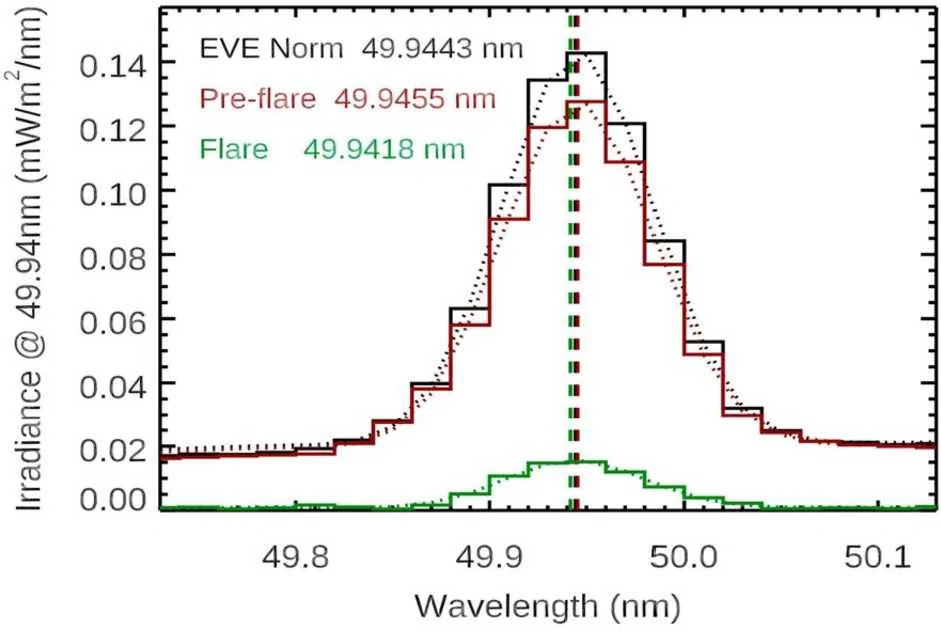
Example spectral feature fit for the Si XII 49.94 nm emission. The EVE Norm (black) is the normal EVE (uncorrected for pre-flare spectrum) spectrum at the flare peak time of 2:02 UT on 2011/046. The Pre-flare (red) is the pre-flare EVE spectrum at 1:20 UT on 2011/046. The Flare spectrum (green) is the EVE-Norm spectrum minus the Pre-flare spectrum. The measurements are the solid lines, and the Gaussian model fits are the dotted lines. The vertical dashed lines are the wavelength center values from the Gaussian fits.

A new EVE data product was developed in 2024 – 2025 and released in July 2025 to provide useful Doppler velocities of 70 different solar EUV lines observed by EVE MEGS. This product, called the EVE Level 4 Lines data product, fits a Gaussian to the spectral feature of interest plus two additional Gaussians for adjacent lines (blends) in the wings of the feature of interest along with a linear background. The Gaussian fit for the feature of interest provides fitted results for the wavelength center, spectral width, and intensity. The two wing-line Gaussian fits only allow adjustment of the intensity for those adjacent lines; that is, the wavelength centers and line widths are fixed for the wing lines. The wavelength centers for the two wing lines were determined initially by fitting Gaussian functions to all of the emission features seen in a MEGS reference spectrum (EVE Level 3 daily average for 5 May 2010). The line width for the two wing lines is 0.05 nm, which is average of the line widths for unblended spectral features in the MEGS spectra. The Gaussian function parameters for all three lines for each spectral feature of interest are listed in the LINE_FIT parameter in the EVE Level 4 Lines product. While a Poisson line function is slightly more accurate for the MEGS spectral line shape, the Gaussian line function proved to be more robust for fitting the many different spectral features and over a wide range of solar activity. In addition, the EVE Level 4 Lines algorithm does additional data analysis for flare events detected in the GOES XRS time series. For those detected flares, a pre-flare MEGS spectrum is subtracted from the MEGS spectra during the GOES flare period to isolate the “flare spectra”, and then the same spectral feature fitting algorithm (three Gaussians and linear background) is used to fit the flare spectra. The FLARE_FLAG in the EVE Level 4 Lines product indicates when the additional fits are done for the “flare spectra”. Figure 1 provides an example spectral fit result for the EVE Level 4 Lines algorithm. This figure shows the spectral feature fits at the flare peak time (EVE Norm, black), the pre-flare time (Pre-flare, red), and a re-fit at the flare peak time as the Flare spectrum (green), which is the EVE-Norm spectrum minus the Pre-flare spectrum. The EVE-Norm wavelength shift from the Pre-flare wavelength corresponds to a Doppler velocity of − 7.2 km/s, and the Flare wavelength shift (relative to the Pre-flare wavelength) corresponds to a larger Doppler velocity of − 22 km/s. The flare spectra Doppler velocities are expected to be larger than the EVE-Norm spectra result because the EVE-Norm spectra include emissions from over the full solar disk; whereas, the flare spectrum usually represents the spectrum from the flaring region only.

Equation 1 provides the conversion to Doppler velocity using the fitted center wavelength values, and where c is the speed of light. Because of small systematic offsets in MEGS wavelength scale and potential for contributions in blending from weaker lines, it is important to use the measured pre-flare center wavelength value instead of theoretical (reference) wavelengths, such as from the CHIANTI spectral model (e.g., Dere et al. 1997). Positive Doppler velocity values are red shifts that indicate line-of-sight flows away from the viewer, or downflow for flare events near the center of the solar disk. Negative Doppler velocities are blue shifts that indicate line-of-sight flows towards the viewer, or upflows for flares near disk center.

1 $$\begin{aligned} \mathrm{V}_{\mathrm{Doppler}}\mathrm{(km/s)} = (\lambda_{\mathrm{flare}}\mathrm{(nm)} - \lambda _{\mathrm{pre}}\mathrm{(nm)}) * \mathrm{c}\mathrm{(km/s)} / \lambda _{\mathrm{pre}}\mathrm{(nm)} \end{aligned}$$

**Figure 2:**
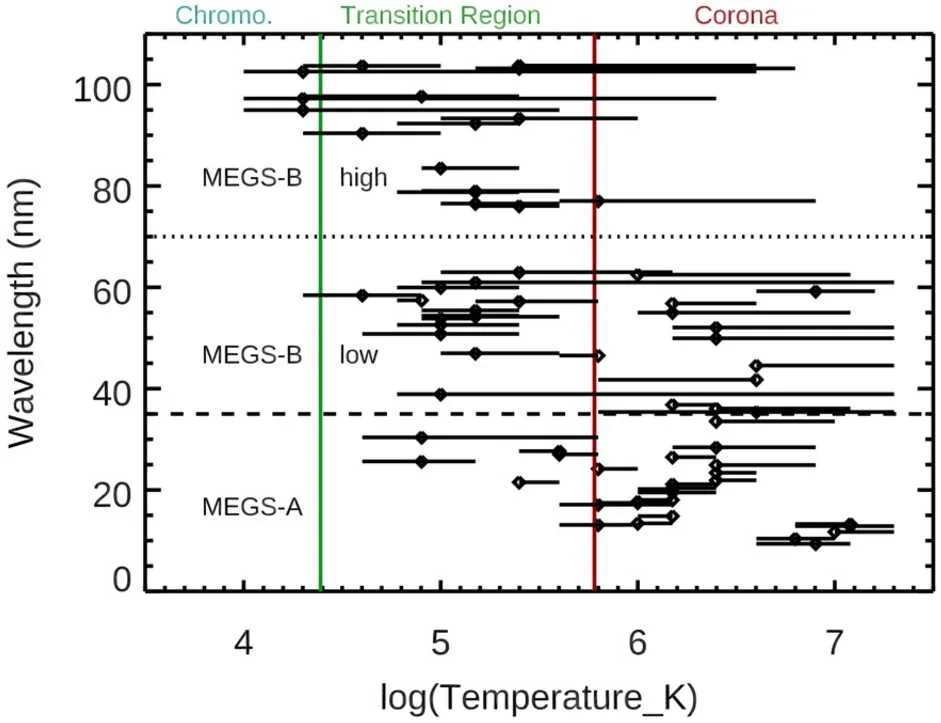
The formation temperatures for the spectral features in the EVE Level 4 Lines product are primarily from the Transition Region (25,000 K to 0.6 MK) and Corona (> 0.6 MK). The diamonds represent the temperature for peak intensity, and the horizontal lines indicate the range of plasma temperature for each spectral feature. The horizontal dashed line separates the MEGS-A and MEGS-B channel ranges. MEGS-A lines cover the corona and hot corona emissions the best. MEGS-B lines cover the transition region and corona temperature ranges well. The horizontal dotted line separates the low and high wavelength ranges for the MEGS-B channel.

There are many different spectral lines in the EUV range that are emitted primarily from the transition region and corona. Many of these emission lines are blended for MEGS spectral resolution of 0.1 nm, so we first analyzed hundreds of the emission features in the MEGS-A and MEGS-B spectra to identify the best emission features to include in the EVE Level 4 Lines product. The 70 spectral features in the EVE Level 4 Lines product were selected based on being unblended (or limited blends) or being a bright feature that is desired for research by the solar physics and terrestrial upper atmosphere communities. Those 70 spectral features are shown as a function of formation temperature in Figure 2 and listed in Table 1. About half of those lines are from the transition region and about half are from the corona. There are also a few lines with contributions from the hot corona (> 6 MK). Due to the MEGS-A CCD capacitor failure in 2014, the MEGS-A lines in the 6 – 37 nm range are only available from May 2010 to June 2014. The MEGS-B spectral measurements are available over the full SDO mission, but they have been limited to a few hours per day due to reduced observations from unexpected degradation in the 70 – 106 nm range during SDO early-orbit operations. The MEGS-B spectra for wavelengths shorter than 70 nm have much less degradation, and thus they have the best measurement precision for the spectral lines in the MEGS-B “low” wavelength range of 33 nm to 70 nm. EVE’s flight software was updated in October 2015 to optimize MEGS-B flare measurements. This update includes new on-board algorithms to autonomously detect the start of a flare and then begin a 3-hour flare campaign for the MEGS-B channel whenever the EVE ESP 1 – 7 nm irradiance goes above a M1 flare level (Woods et al. 2026).

**Table 1:**
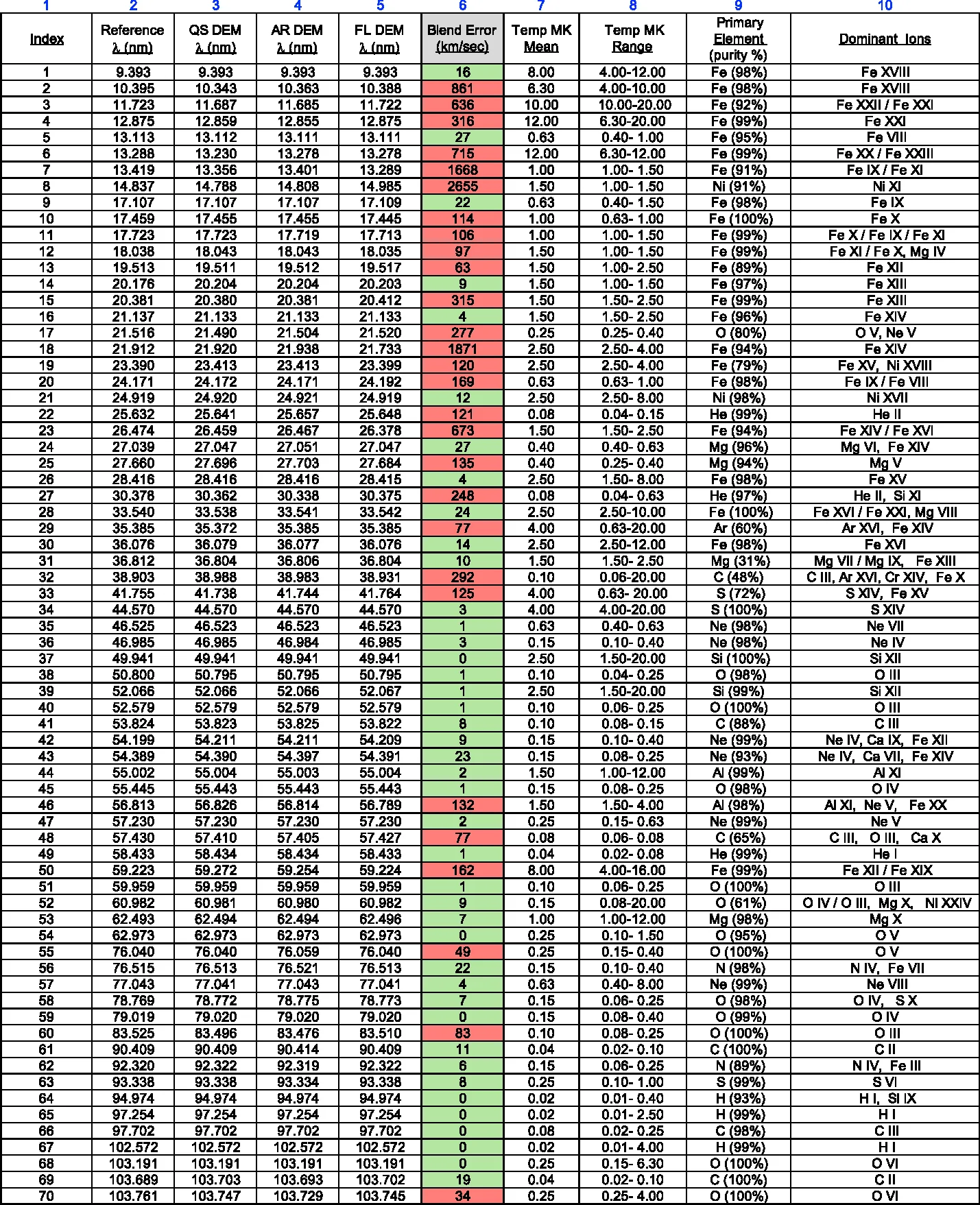
List of 70 Spectral Features in EVE Level 4 Lines Data Product. The 42 lines with a Blend Error less than 30 km/s and in green highlight are considered the best lines to study flare dynamics.

The CHIANTI spectral model was used in two different ways to determine the line blends for each spectral feature. One method involved analysis of the line lists from the CHIANTI model for iso-thermal spectra with plasma temperature ranging from 10,000 K to 20 MK with five temperatures per order of magnitude change in temperature. This type of analysis identified the line contributions at MEGS 0.1-nm spectral resolution as a function of plasma temperature, yielding complicated tables of line blends at each plasma temperature. Because the actual solar EUV spectrum is not well represented by a single plasma temperature, this iso-thermal line-blend analysis was not as revealing about line blends over solar activity as the second line-blend analysis. Example tables of this type of iso-thermal line-blend analysis are shown in Appendix A of Woods et al. (2023).

The second line-blend analysis examined the CHIANTI spectral model line lists for three different reference differential emission measure (DEM) spectra. Those reference spectra cover a wide range of plasma temperature and are generated using the Quiet Sun (QS), Active Region (AR), and Flare DEM options in the CHIANTI model. In addition to identifying the line blends for each spectral feature for the three DEM spectra, we analyze those DEM spectra with our Level 4 Lines fit algorithm (three Gaussians and linear background) to determine the center wavelength for their spectra features. Then the maximum spread in the fitted center wavelengths between those three DEM results provides an estimate for the spectral shifts over the expected range of solar activity (QS, AR, FLARE). This maximum spread in center wavelength is considered to be an indicator for line blend uncertainties as the solar atmosphere temperature changes with solar activity. This maximum spread in nm is converted to Doppler velocity units of km/s and listed in the Blend Error column in Table 1.

As can be seen in the Table 1 listing, there are many blended lines that can have large wavelength shifts with changing solar activity, and thus those spectral features with a Blend Error exceeding 30 km/s are considered not useful for flare Doppler velocity analysis. In other words, those blended lines have large wavelength shifts for the 0.1-nm-resolution MEGS spectral features due to different emission lines dominating at different plasma temperatures. There are 42 spectral features (9 for MEGS-A and 33 for MEGS-B) that have their Blend Error estimate below 30 km/s, and those spectral features are considered the best for studying the flare dynamics, as discussed later in Section 4.

## MEGS Optical Wavelength Shifts

While the original MEGS optical design did not have a requirement to measure solar Doppler velocities, the fundamental MEGS optical design characteristics result in observable optical wavelength shifts that are sensitive to the location of active regions (flares) on the solar disk. These optical wavelength shifts for MEGS-A are discussed in detail by Chamberlin (2016), so the MEGS-B optical wavelength shifts are the primary focus for this section.

**Figure 3:**
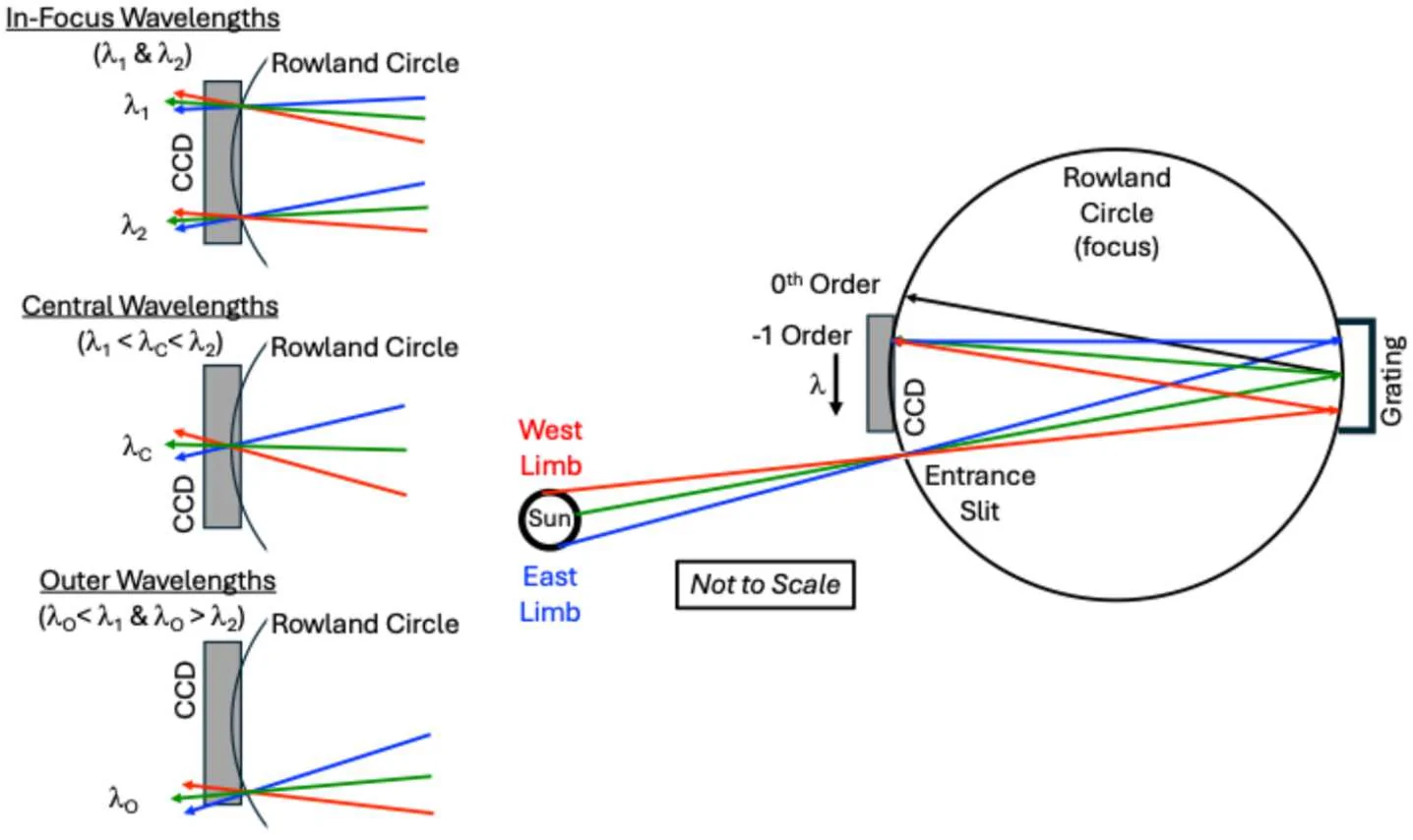
Cartoon of the focal properties for a Rowland-circle spectrograph. The flat CCD sensor can be placed on the Rowland circle to have two in-focus wavelengths ($\lambda _{1}$ and $\lambda _{2}$). The central wavelengths ($\lambda _{1}< \lambda _{\mathrm{C}}< \lambda _{2}$) have optical wavelength shifts for active regions not at solar disk center because the CCD plane is in front of the Rowland circle. The outer wavelengths ($\lambda _{\mathrm{O}}< \lambda _{1}$ and $\lambda _{\mathrm{O}}> \lambda _{2}$) also have optical wavelength shifts for active regions not at solar disk center, but the outer wavelengths have their east-limb and west-limb shifts flipped relative to the central wavelengths shifts because the CCD plane is behind the Rowland circle for the outer wavelengths. The green ray is for disk center. Blue ray is for east-limb, and red ray is for west-limb. The MEGS-B optical design is more complicated as it has two Rowland-circle spectrographs in tandem.

The MEGS-B optical design (Crotser et al. 2007; Woods et al. 2012) has two Rowland-circle spectrographs with the first one to limit the spectral passband of 33 – 107 nm into the second spectrograph. Those two gratings have their grating grooves rotated 90° to each other to help isolate (remove) higher order contributions. Therefore, the MEGS-B spectrum for grating order of − 1 from both spectrographs is illuminated diagonally across the MEGS-B CCD sensor. The best spectral focus for a Rowland-circle spectrograph is along the Rowland circle, but only two wavelengths can be in near-perfect focus on the Rowland circle because the MEGS CCD is a flat sensor. Consequently, all other wavelengths will be slightly out of focus and thus the wavelength scale can be sensitive to active region (flare) location. Namely, if the CCD sensor plane is slightly in front of the Rowland circle for a specific wavelength (see Figure 3 central wavelength case), then the east-limb active regions will have slight wavelength shift from the spectral contribution from the active regions at disk center and west-limb active regions will also have slight wavelength shift, but in opposite direction from the east-limb active region spectrum. Similarly, if the CCD sensor plane is slightly behind the Rowland circle for a different wavelength (see Figure 3 outer wavelength case), there will be similar wavelength shifts but with a flip in wavelength shift direction for the east-limb and west-limb active regions relative to the case of the CCD plane being in front of the Rowland circle.

**Figure 4:**
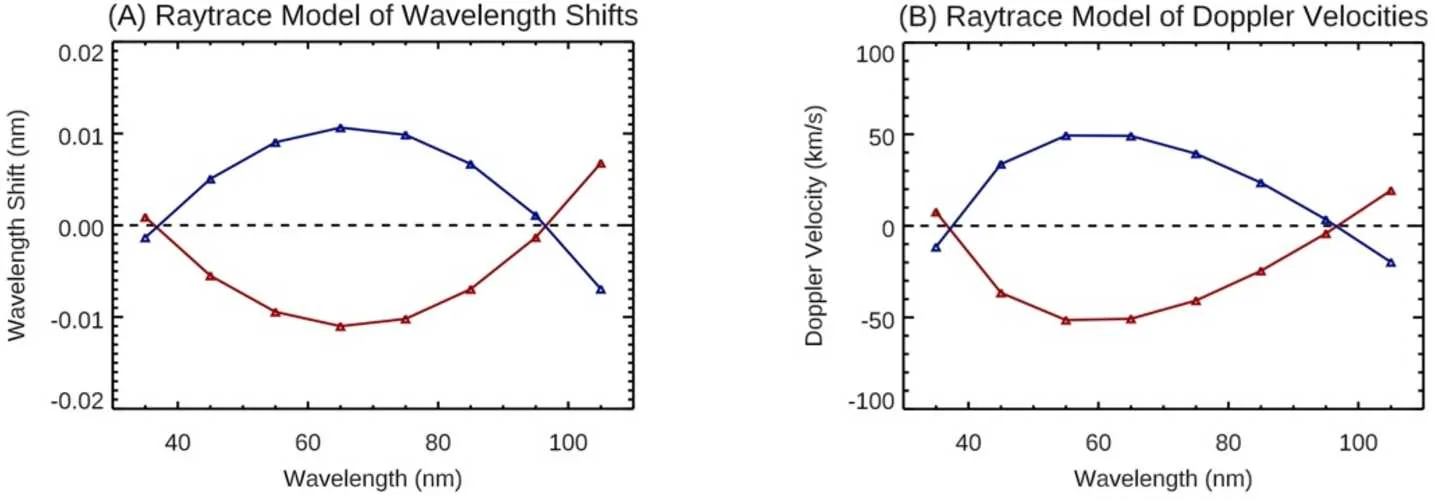
MEGS-B original-design raytrace results for active regions (flares) near the east and west solar limbs. There are two near-perfect-focus wavelengths near 30 nm and 100 nm where there are no optical wavelength shifts with active regions on the limbs, but all other wavelengths have some optical wavelength shifts dependent on the active region (flare) location. The left panel shows the wavelength shift in units of nm, and the right plot converts those wavelength shifts to Doppler velocities. The east-limb (red, − 15 arc-min) and west-limb (blue, + 15 arc-min) results are relative to the wavelength when the active region is at disk center.

This simple explanation for the optical wavelength shifts for a Rowland-circle spectrograph is further complicated for MEGS-B because there are two Rowland-circle spectrographs in MEGS-B. Raytrace results of the original MEGS-B design, as shown in Figure 4, provide the expected optical wavelength shifts as a function of wavelength and as a function of active region location. Figure 4 shows the wavelength shifts and the conversion of those wavelength shifts to Doppler-equivalent velocities, and those are relative to the wavelength when the active region is at disk center. Equation 1 is used for this Doppler velocity conversion with its pre-flare wavelength (λ_pre_) replaced with the disk-center wavelength ($\lambda _{\mathrm{C}}$). These MEGS-B raytrace results show very similar results as single Rowland-circle spectrograph results in that (1) there are two wavelengths (near 30 nm and 100 nm) with very small (no) optical wavelength shifts because they are in focus at the Rowland circle, (2) the central wavelengths between these two near-perfect-focus wavelengths have modest optical wavelength shifts because those wavelengths illuminate the CCD before the Rowland circle, (3) the outer wavelengths outside of these two near-perfect-focus wavelengths have modest optical wavelength shifts because those wavelengths illuminate the CCD after the Rowland circle, and (4) the optical wavelength shifts are flipped in direction between the central wavelengths and outer wavelengths in regards to east-limb versus west-limb active regions (flares).

Because the SDO spacecraft always keeps the solar north pole fixed relative to MEGS-B optical axis, these raytrace results are applicable over the entire SDO mission. We note that for solar observations from a low Earth orbit (LEO) satellite, where the solar north pole rotates during an orbit relative to the solar instrument optical axis, the optical wavelength shift would also have to consider the angle between the solar north pole relative to the instrument optical axis.

**Figure 5:**
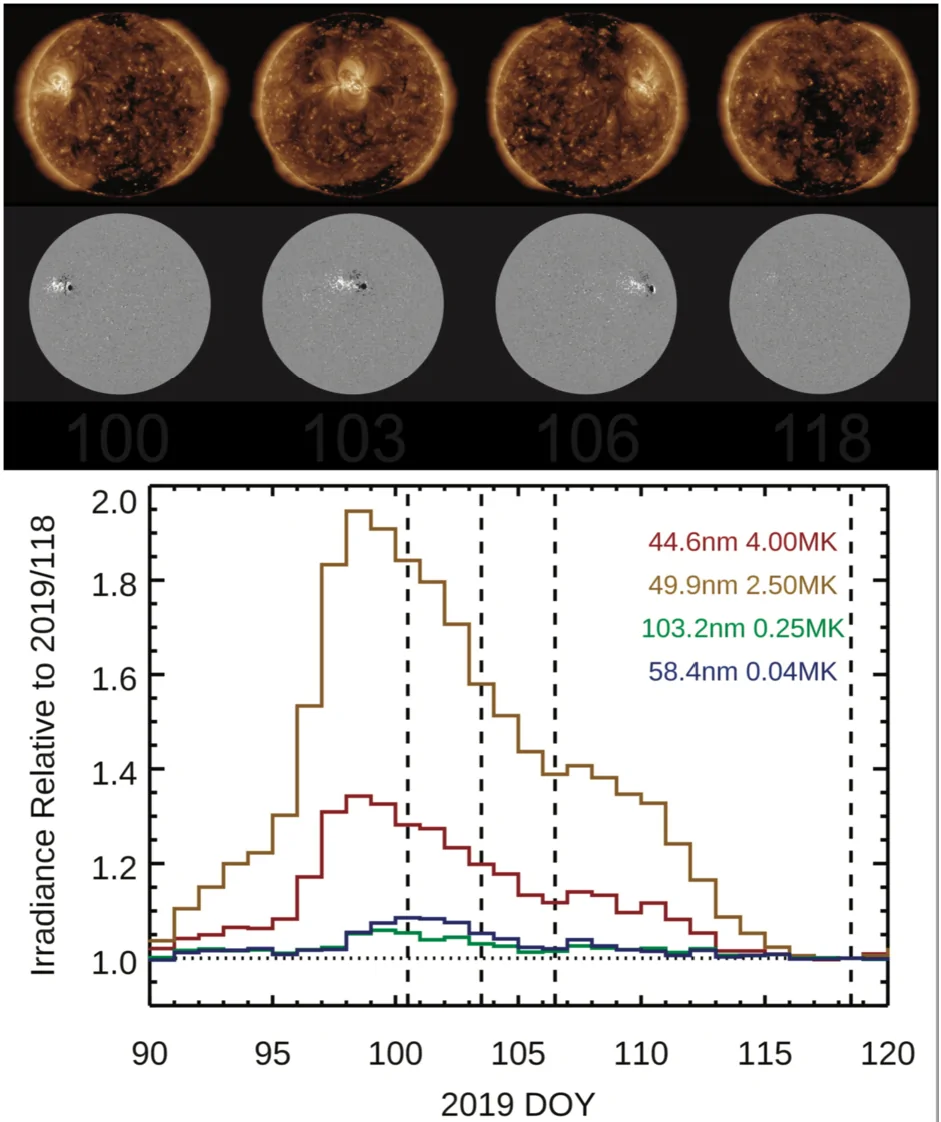
MEGS solar EUV irradiance time series (bottom) and HMI B_LOS_ magnetograms (middle) and AIA 193 Å solar images (top) are shown for April 2019. A single active region was on the solar disk between 10 April (DOY 100) and 16 April (DOY 106), and this period is used to validate the raytrace model of MEGS-B optical wavelength shifts as function of active region location. The MEGS time series is normalized to the spectral irradiance on spotless day 28 April (DOY 118). The four vertical dashed lines correspond to the time of the solar images.

To validate these raytrace results, one can examine the wavelength shift results during a solar rotation time period when only one primary active region is on the solar disk. Finding a time period with a single active region on the solar disk over several days is actually very challenging because it is rare to have just one bright active region on the disk and because active regions evolve significantly over a few days. The raytrace validation period that we have identified is 10 April 2019 to 16 April 2019 as shown for EVE time series and SDO solar images (e.g., Lemen et al. 2012) in Figure 5. This period is near the last solar cycle minimum that occurred in late 2019 for most EUV wavelengths. This period has the NOAA active region number 12738 starting out on 10 April 12UT at N06-E38 and ending on 16 April 12UT at N06-W42. This active region is closest to disk center at about 8 UT on 13 April. Extending this time window to enable this active region to be closer to the limb is problematic because there is a west-limb active region (NOAA-12737) on 9 April and earlier, and a new active region appears east of the NOAA-12738 active region starting on 17 April.

**Figure 6:**
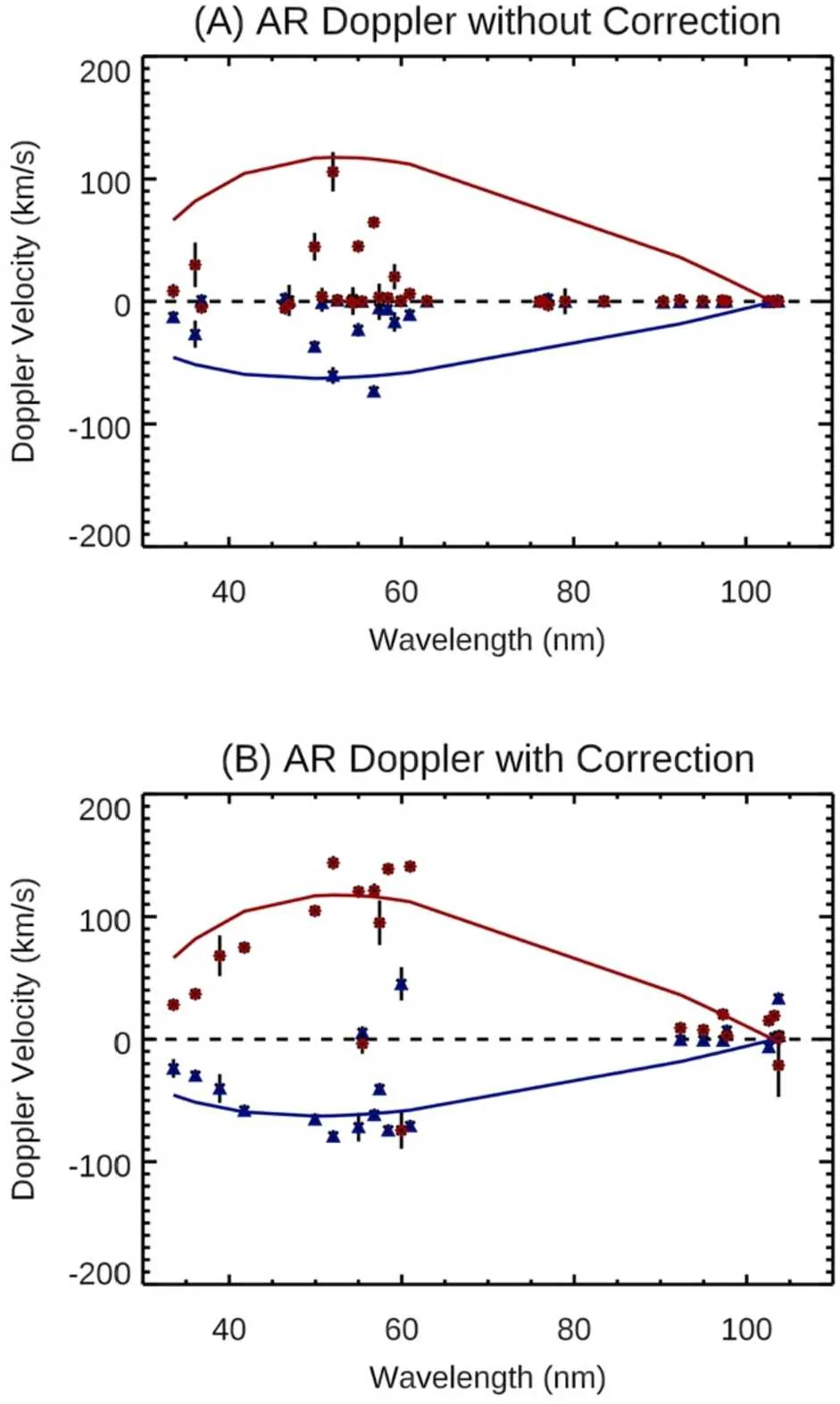
Measured MEGS-B wavelength shifts for the single active region rotation in April 2019 are converted to Doppler velocities. The red and blue velocities are for positive and negative wavelength shifts, respectively. The MEGS-B tuned raytrace model results are the solid lines and include a eastward offset of 2.5 arc-min to account for MEGS-B misalignment from Sun center. Panels A and B show Doppler velocities without and with the spotless-day correction, respectively. Correction for the solar full-disk contribution is critical for this comparison to the MEGS-B raytrace model.

The measured wavelength shifts between 10 April 2019 (38°east) and 13 April 2019 (center) and between 16 April 2019 (42°west) and 13 April 2019 (center) have somewhat confusing results when examining the wavelength shifts for the full-disk irradiance as shown in Figure 6A. In particular, the coronal lines have larger wavelength shifts than the transition region lines. The solar full-disk irradiance can be a significant contribution to the MEGS solar EUV irradiance relative to the contribution from the single active region, especially so for chromospheric and transition region emissions versus the more variable coronal emissions as illustrated in Figure 5. Therefore, the MEGS-B spectrum from the spotless day of 28 April 2019 is first subtracted from the EVE Level 3 (daily average) solar EUV irradiance before applying the Level-4-Lines line-fitting algorithm for the solar spectra on 10 April, 13 April, and 16 April. This subtraction (correction) of the spotless-day spectrum effectively removes the solar full-disk irradiance contribution to provide a reasonable estimate for the “active-region spectra”. Those corrected spectra results are shown in Figure 6B and indicate much more consistent wavelength shifts for both corona and transition region emission lines once this spotless-day correction has been made. The few out-of-family wavelength shifts are possibly related to larger-than-expected line blends for those lines.

One will notice that these measured wavelength shifts for this April 2019 active region as shown in Figure 6B are not symmetrical about zero. Figure 4 shows east-west symmetrical offsets from zero because the MEGS-B original-design raytrace model assumed that the Sun center is at the MEGS-B optical center. Because the pre-flight EVE alignment to the SDO spacecraft was performed as a compromise between the different optical centers of the MEGS-A, MEGS-B, and ESP channels, plus any optics shifts from launch vibrations, the actual Sun center is not at the MEGS-A optical center. This east-west asymmetry in the measured wavelength shifts (Figure 6B) suggests that there is a eastward offset of 2.5 arc-min to account for MEGS-B misalignment from Sun center. We note that the EVE channels all have large fields of view of about 2 degrees and that the EVE-spacecraft alignment requirement was to be within 6 arc-minutes of Sun center.

The MEGS-B original-design raytrace model (Figure 4 result) was adjusted to better match the wavelength shifts of the active-region spectra during April 2019. The adjustments to the initial MEGS-B raytrace model to best fit the measurements shown in Figure 6B include (1) 2.7 arc-min eastward offset of Sun center from optical center (slightly more than the 2.5 arc-min derived from the measured shifts), (2) moving grating #1 by 0.14 mm closer to entrance slit, (3) moving grating #2 by 0.11 mm closer to grating #1, (4) moving CCD sensor by 0.10 mm closer to grating #2, and (5) rotating CCD sensor by 0.1° relative to design position. The wavelength shift results from this tuned raytrace model are plotted in Figures 6A and 6B as the solid lines. These results indicate good agreement between the measured and modeled optical wavelength shifts only if the spotless-day corrections are made (Figure 6B). If the spotless-day correction is not made (Figure 6A), then only the coronal emission lines have wavelength shifts similar to the MEGS-B tuned raytrace model.

The Figure 6A result helps to explain why the coronal emissions in the prograde-rotation study of Hudson et al. (2022) and Fitzpatrick and Hudson (2022) have larger wavelength shifts than the transition region emissions in their study. Furthermore, these raytrace modeling results of the MEGS-B optical wavelength shifts indicate that the prograde-rotation results of Hudson et al. (2022) and Fitzpatrick and Hudson (2022) are not actually solar based but are the consequence of MEGS-B optical wavelength shifts as a function of active region location.

The April-2019 measured wavelength shifts (shown as Doppler velocities in Figure 6B) are fitted with a parabolic function (Equation 2A – 2C), and these related parabolic function parameters as a function of wavelength can be used for correcting the optical wavelength shifts for the EVE L4L spectral lines with indices between 28 and 70 (MEGS-B wavelengths). This correction involves using the line’s wavelength in Equation 2A to get its $\Delta \lambda $_limb_ value, then using the AR (or flare) location (LONG, LAT in Stonyhurst heliographic coordinates) in Equation 2C to get the AR offset X_AR_ value, and finally using those two calculated values in Equation 2B to get the predicted optical wavelength shift, $\Delta \lambda $. The parameter values for Equation 2A are a$_{\mathrm{B}} = -0.07092\text{ nm}$, $\mathrm{b}_{\mathrm{B}} = 0.003062$, and $\mathrm{c}_{\mathrm{B}} = -2.338\times10^{-5}\text{ nm}^{-1}$. The R_Sun_ value at 1 AU is 16 arc-min. The X_offset_ value is − 2.5 arc-min, which means that the MEGS-B optical axis has eastward offset from Sun center. For flare analysis with a known location of the flare on the solar disk, we recommend the use of Equation 2A – 2C to correct MEGS-B optical wavelength shifts. These optical wavelength shifts for an east limb active region (flare) are negative for most wavelengths, and the optical wavelength shifts for a west limb active region (flare) are positive for most wavelengths (see Figure 6).

2A $$\begin{aligned} &\Delta \lambda _{\mathrm{limb}}\mathrm{(nm)} = \mathrm{a}_{\mathrm{B}} + \mathrm{b}_{\mathrm{B}} * \lambda \mathrm{(nm)} +\mathrm{c}_{\mathrm{B}} * \lambda ^{2}\mathrm{(nm}^{2}\mathrm{)} \end{aligned}$$

2B $$\begin{aligned} &\Delta \lambda \mathrm{(nm)} = \Delta \lambda _{\mathrm{limb}}\mathrm{(nm)} * \mathrm{X}_{\mathrm{AR}}\mathrm{(arc-min)} / \mathrm{R}_{\mathrm{Sun}}\mathrm{(arc-min)} \end{aligned}$$

2C $$\begin{aligned} &\mathrm{X}_{\mathrm{AR}}\mathrm{(arc-min)}= \mathrm{R}_{\mathrm{Sun}}\mathrm{(arc-min)} * \sin\mathrm{(LONG)} * \cos\mathrm{(LAT)} - \mathrm{X}\underline{\mathrm{offset}} \end{aligned}$$

**Figure 7:**
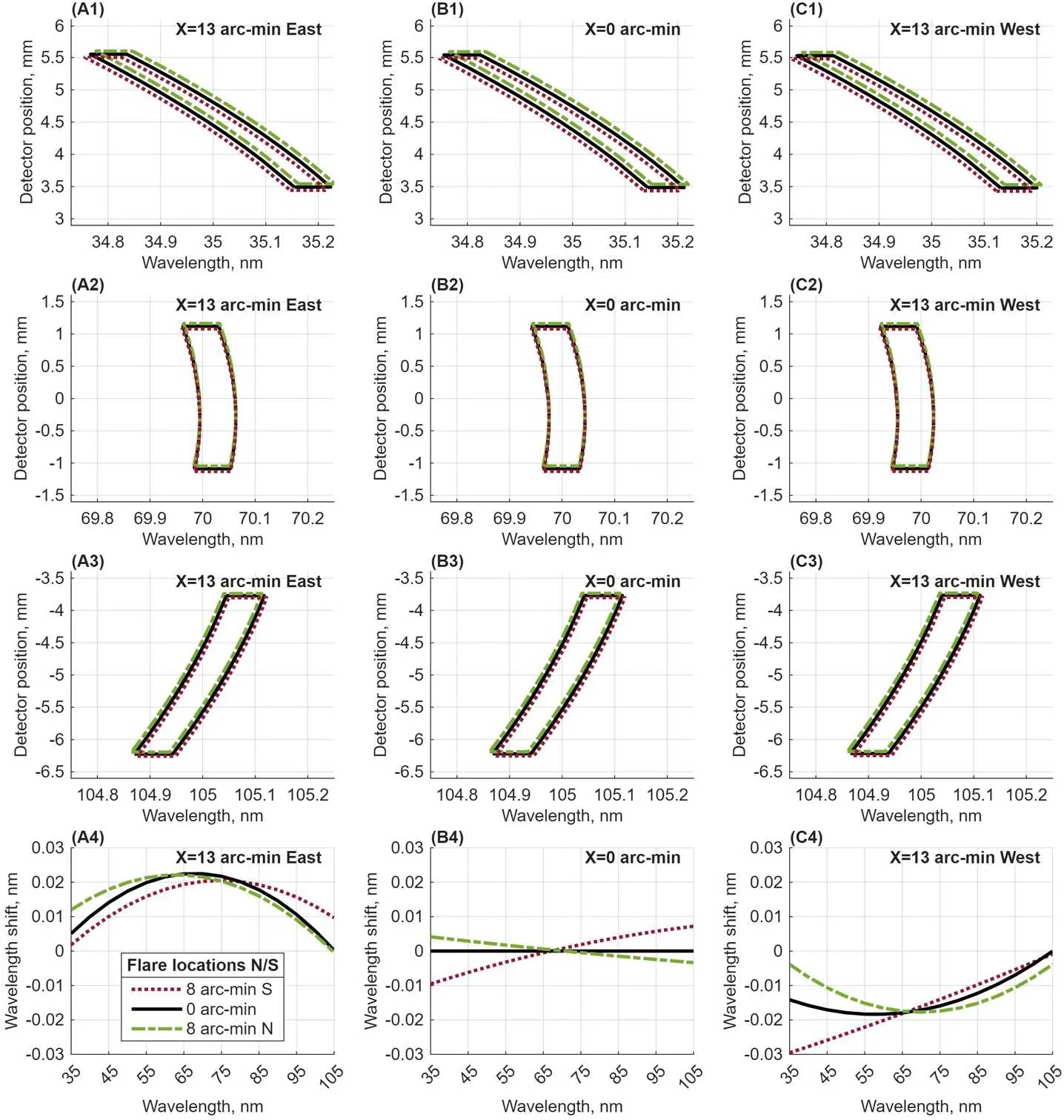
The estimated MEGS-B optical wavelength shifts as function of latitude are shown using the tuned raytrace model for three different longitudes: (A) − 13 arc-min X offset (east), (B) zero X offset, and (C) + 13 arc-min X offset (west). For each of these X offsets, there are three different latitudes with Y offsets of − 8 arc-min (south, red), 0 (center, black), and + 8 arc-min (north, green). The three different X offsets and three different Y offsets represent a total of nine different active region (flare) locations used for this raytrace analysis. The top 9 plots show the raytrace model spot diagram for three wavelengths of 35 nm, 70 nm, and 105 nm. The three bottom plots (A4, B4, C4) show the estimated wavelength shifts for those nine different active region locations.

Because the MEGS-B slit images onto the CCD sensor are curved, there is also a smaller optical wavelength shift as related to when the active region (flare) latitude is not at the solar equator. The curvature of the slit images at each wavelength is mainly due to astigmatism of the concave gratings, so the amount of wavelength shift is dependent on the latitude. The April 2019 solar rotation measurements worked well to characterize the X-axis (solar longitude) optical wavelength shifts for MEGS-B but are not useful to characterize the Y-axis (solar latitude) optical wavelength shifts. Therefore, the MEGS-B tuned raytrace model is used to estimate the worst-case optical wavelength shift for the MEGS-B Y-axis by modeling an active region (flare) at ± 8 arc-min from solar equator (that is, at solar latitude of ± 30°). Those raytrace results are shown in Figure 7 for nine different active region (flare) locations. Figure 7 includes raytrace model spot boundaries (curved slit images) and is organized with left panels (A1-A4) having X offset of − 13 arc-min (east), middle panels (B1-B4) having X offset of 0 (optical center), and right panels (C1-C4) having X offset of + 13 arc-min (west). These all are based on the MEGS-B tuned raytrace model that has additional − 2.5 arc-min X offset of MEGS-B optical center from Sun center, so the black lines in the Figure 7 bottom panels (A4, B4, C4) are similar to the Figure 6 raytrace model results but with this − 2.5 arc-min X offset already applied for Figure 7 results. We also note that Figure 7 shows wavelength shifts while Figure 6 shows Doppler velocities. The dotted red lines and dashed green lines in Figure 7 bottom panels are the estimated optical wavelength shifts for Y offsets of − 8 arc-min (south) and + 8 arc-min (north), respectively. A key conclusion from this Y-offset raytrace model analysis is that the optical wavelength shifts from Y offsets (solar latitude) are about one third of the sensitivity of the X-offset (solar longitude) wavelength shifts. Therefore, the following discussions about flare Doppler velocities may have corrections to the optical wavelength shifts related to solar longitude (X-axis, Equation 2A – 2C) but no corrections for solar latitude (Y-axis) have been applied.

For the other EVE L4L spectral lines with indices between 1 and 31 (MEGS-A wavelengths), corrections for the MEGS-A optical wavelength shifts as function of active region and flare location need to be made prior to Doppler velocity analysis. In particular, flares are projected differently onto the MEGS-A detector depending on their location on the solar disk. This MEGS-A sensitivity to flare location results in unique wavelength scale changes that must be made for each flare when using the EVE Level 0B data product (MEGS raw images without any corrections). These images have shown to produce useful science but must be corrected with optical wavelength shifts and for degradation trends. A paper in preparation by G. Gonzalez and P. Chamberlin will describe these detailed MEGS-A corrections for using the EVE Level 0B images and also will provide improved analyses of Doppler velocities over the lifetime of several flares. For now, the MEGS-A optical wavelength shifts and related equations are provided for a few wavelengths in Chamberlin (2016) and Gonzalez, Chamberlin, and Herde (2024).

## Doppler Velocity Results During Solar Flares

As mentioned already, the solar Doppler velocity results most useful with the new EVE Level-4 Lines data product are for flare events. The larger flares, above about M5, have enough flare intensity to provide good measurement precision of the flare variability and associated Doppler wavelength shift in the EVE Level 4 Lines data product. For the smaller flares, the amount of flare variability is only a couple percent for most EUV features, which is comparable to the MEGS measurement precision for individual spectra. The EVE Level 4 Lines product does use 1-min averaged spectra, versus the individual 10-sec spectra, to help improve the measurement precision by about a factor of two. The limited exposure time for MEGS over much of the SDO mission has reduced the number of X-class flares observed by MEGS. In particular, MEGS-A only observed in 2010 – 2014, and MEGS-B observations are limited to a few hours per day. Furthermore, there was weaker solar activity between 2010 and 2021 for solar cycle 24. Nonetheless, there are 42 X-class flare observations for MEGS-A and 103 X-class flare observations for MEGS-B. The number of coincident X-class flares for both MEGS-A and MEGS-B is just 19. As discussed in Section 2, it is critical to subtract a pre-flare spectrum from a MEGS spectrum to obtain a “flare spectrum”. That correction is included in the EVE Level 4 Lines data product for most of the C-X class flares observed by MEGS. Not all flare events have this correction because the EVE Level-4-Lines processing algorithm, which examines the GOES XRS time series data to identify the times of flare events, is not designed to detect flares smaller than C class and does not usually detect slowly evolving flares (e.g., some filament eruptions). This section shows some examples of Doppler velocity results during some of those X-class flares.

**Figure 8:**
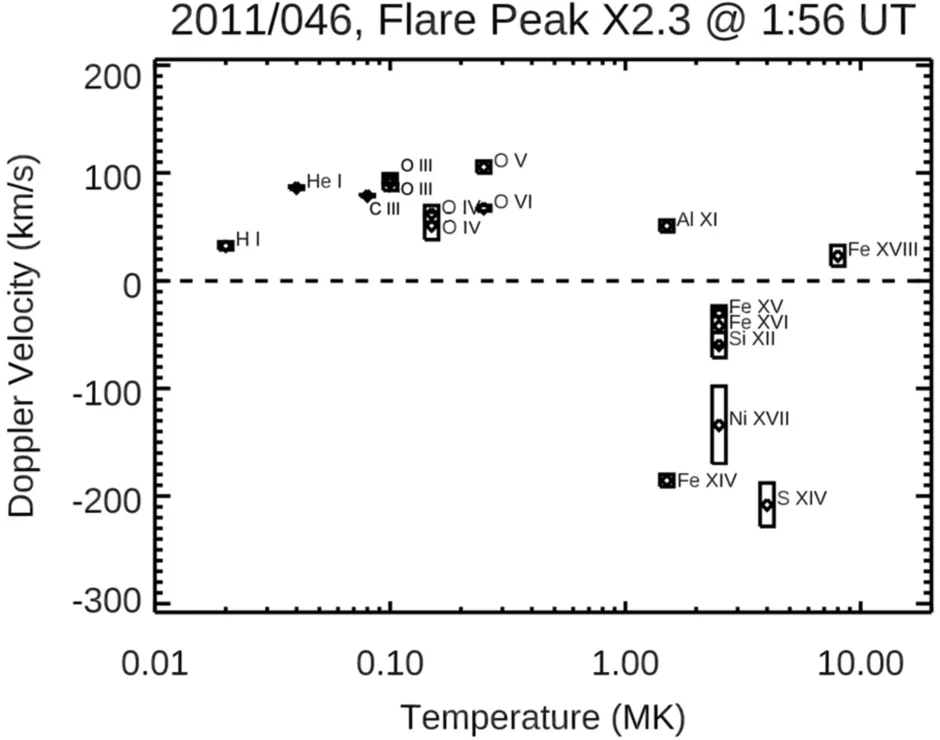
Maximum Doppler velocities during the X2.2 flare on 15 February 2011 as a function of line formation temperature. These maximum Doppler velocities occurred during this flare’s impulsive phase. The rectangles are error bars that are the uncertainties for the fitted wavelength shifts converted to Doppler velocity units.

The first X-class flare during the SDO mission was the X2.2 flare on 15 February 2011 with GOES XRS flare peak time of 1:56 UT and location of S22-W13 on the solar disk. Woods et al. (2026) present that flare’s Doppler velocity results from the EVE Level 4 Lines data product, so only a brief summary is provided here for that flare example. It is important to note that the “low-error” spectral features in the EVE Level 4 Lines data product are those listed in Table 1 with Blend Error less than 30 km/sec. As noted in Section 2, the “flare Doppler velocities” are derived as the flare spectrum wavelength minus the pre-flare spectrum wavelength (Equation 1). The maximum Doppler velocities for the “low-error” spectral features as a function of line formation temperature are shown in Figure 8 for this X2.2 flare. One conclusion is that the time of maximum Doppler velocity is during the flare’s impulsive phase for most spectral features. All of the chromosphere, transition region, and cool corona emission features with formation temperature less than 1.0 MK have a maximum Doppler velocity that is positive and thus indicating downflow of flare-affected plasma in those solar layers. The average of these positive Doppler velocities is 75±24 km/sec. In contrast, the warmer corona emissions with formation temperature more than 1.0 MK mostly have negative Doppler velocities and thus indicating upflow of flare-affected plasma for an eruptive flare, perhaps even a sign for a coronal mass ejection (CME) event. There isn’t as much consistency for the blue (upward) Doppler velocity values. We note the range of Doppler velocities with largest blueshift by the Fe XIV feature (21.14 nm) of − 213 km/sec to a redshift of + 50 km/sec for the hotter Fe XVIII (9.39 nm) feature. The average of just the negative Doppler velocities is −114±75 km/sec. Woods et al. (2026) present additional time series of the Doppler velocities of select wavelengths during this X2.2 flare and show that there are multiple flare heating periods during this flare that result in additional local maxima of Doppler velocities that are smaller than the first maximum of Doppler velocities during the impulsive phase.

**Figure 9:**
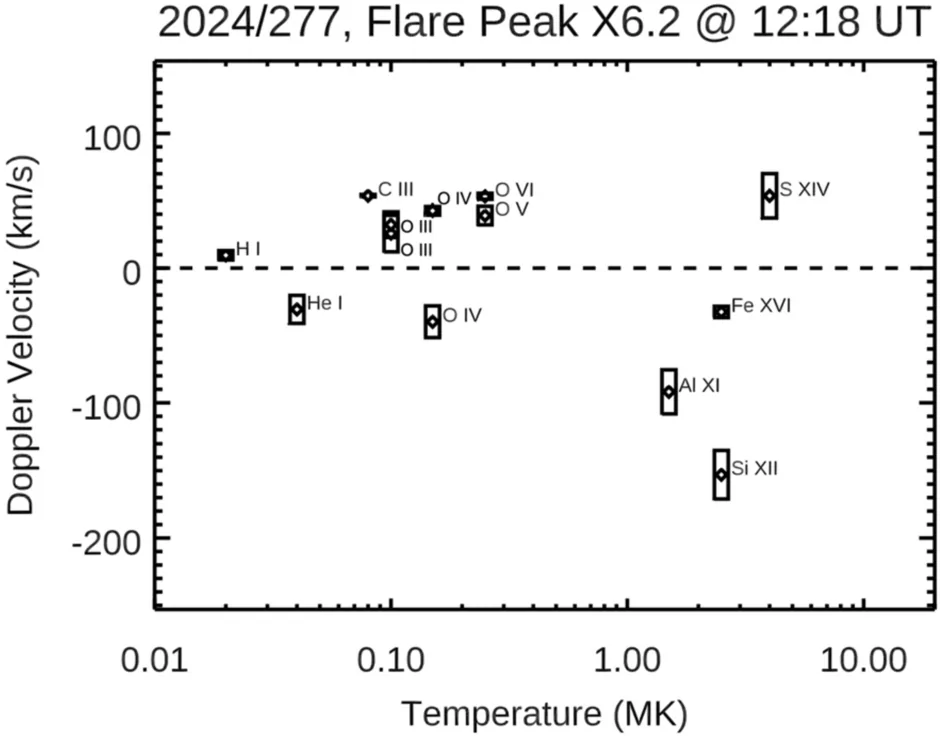
Maximum Doppler velocities during the X9.0 flare on 3 October 2024 as a function of line formation temperature. These maximum Doppler velocities occurred during this flare’s impulsive phase. The rectangles are error bars that are the uncertainties for the fitted wavelength shifts converted to Doppler velocity units.

This Doppler velocity behavior as function of line formation temperature for this X2.2 flare is similar for other EVE-observed flares that occur near disk center. Another example for a flare near disk center is shown in Figure 9 for the X9.0 flare on 3 October 2024 with GOES XRS flare peak time of 12:18 UT and location of S10-W05 on the solar disk. With this flare being later in the SDO mission, there are only MEGS-B lines included in this analysis. The same nine transition region lines from MEGS-B are included in both Figures 8 and 9, but there are only four coronal lines from MEGS-B in Figure 9. The dynamics for this X9.0 flare have similar results as for the X2.2 flare. For one, the time of maximum Doppler velocity during this X9.0 flare is during the impulsive phase for most spectral features. Most of the cooler emission features with formation temperature less than 1.0 MK have a maximum Doppler velocity that is positive (downflow). The average of the positive Doppler velocities is 37±16 km/sec. Additionally, the warmer corona emissions with formation temperature more than 1.0 MK mostly have negative Doppler velocities (upflow). The average of the negative Doppler velocities is −94±58 km/sec. While the X9.0 flare has much larger variability than the X2.2 flare, the Doppler velocity behavior below and above 1.0 MK is similar for both of these flares, and the magnitudes of the Doppler velocities for the X9.0 flare are less than those for the X2.2 flare.

These flare Doppler velocity results from EVE are consistent with flare Doppler velocity expectations as described by Milligan and Dennis (2009) with redshifts for chromospheric and transition-region lines and blueshifts for hot coronal lines. The transition from redshifts to blueshifts between 1 and 2 MK is also noted by Milligan and Dennis (2009). Additionally, these Doppler results for this X2.2 flare are consistent in magnitude of about 50 km/sec to other flare analyses of EVE Doppler velocities by Hudson et al. (2011), Brown, Fletcher, and Labrosse (2016), and Otsu and Asai (2024). In particular, Hudson et al. (2011) shows a redshift of 50 km/sec for He II (30.4 nm) and a blue shift of about 100 km/sec for Fe XXIV (19.2 nm). Brown, Fletcher, and Labrosse (2016) examined the H Lyman lines and C III (53.8 nm) Doppler velocities and found shifts in range of 20 – 50 km/sec with redshifts for three flares and blueshifts for three different flares. As another example, Otsu and Asai (2024) studied the EVE O V (63.0 nm) and O VI (103.2 nm) features during the M8.7 flare on 2 October 2022 and found a large − 400 km/sec blueshift during the filament eruption associated with this flare and then followed by a redshift during the flare decay phase as downflows for the cooling post-flare loops.

Because Doppler wavelength shifts are along the line-of-sight, flares near the solar disk center are expected to have larger Doppler velocities as compared to the flares near the solar limb. If one wants to assume that the Doppler velocities are primarily downflow or upflow plasma movement along the solar radius, then one expects that the Doppler velocities shown in Figures 8 and 9 for flares near disk center to potentially scale down in magnitude as the flares are closer to the limb. It is critical to make the MEGS optical wavelength shift correction (from Section 3) to the flares not at disk center; else one will find large wavelength shifts (50 – 200 km/s) for the limb flares due to MEGS optical design and not related to true solar dynamics.

**Figure 10:**
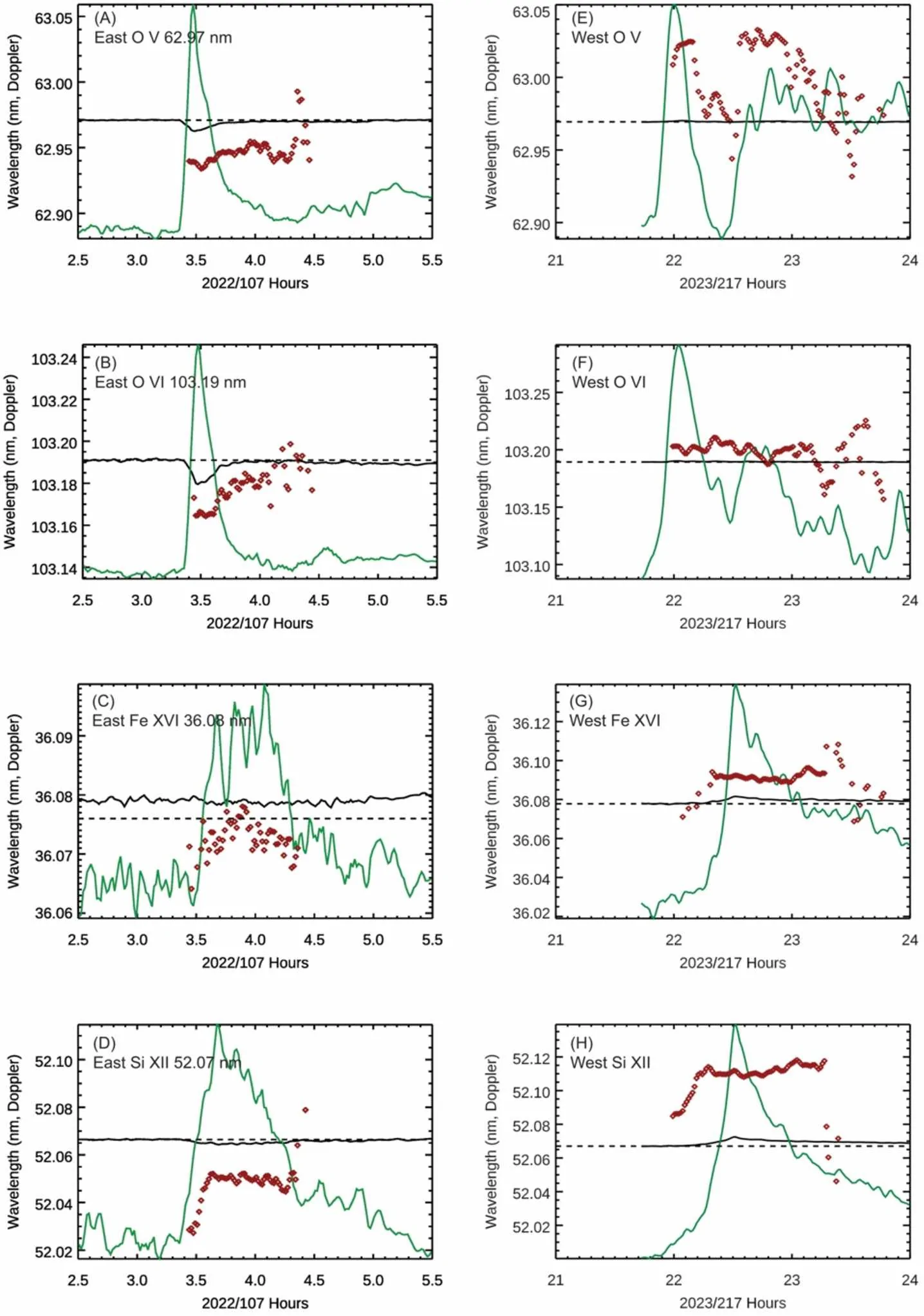
Left plots are wavelength shifts during the east-limb X1.1 flare on 17 April 2022 (location N6-E80), and right plots are wavelength shifts during the west-limb X1.6 flare on 5 August 2023 (location N13-W79). The four emissions features are O V 62.97 nm at 0.25 MK (panels A, E), O VI 103.19 nm at 0.25 MK (panels B, F), Fe XVI 36.08 nm at 2.5 MK (panels C, G), and Si XII 52.07 nm at 2.5 MK (panels D, H). The black lines are the central wavelengths fit to the full-disk irradiance, and the red diamonds are the central wavelengths fit to the flare irradiance (full-disk spectrum minus pre-flare spectrum). The dashed horizontal line is the pre-flare reference wavelength. The green lines are scaled irradiances for each emission feature. The wavelength shifts for the east-limb flare are mostly blue-shifted (negative), and the wavelength shifts for the west-limb flare are mostly red-shifted (positive) because the MEGS-B optical wavelength shift correction (Equation 2A – 2C) was not applied for these plots.

To illustrate that point, Figure 10 shows the wavelength shifts for an east-limb flare on 17 April 2022 and a west-limb flare on 5 August 2023 without making this MEGS optical wavelength shift correction. In particular, the data shown in Figure 10 are directly from the EVE Level 4 Lines data product and so those data are corrected with a pre-flare spectrum but not corrected for optical wavelength shifts related to flare location. Note in Figure 10, the wavelength shifts are mostly blue-shifted for the east-limb flare and mostly red-shifted for the west-limb flare. The east-limb flare suggests large impulsive phase intensity for the transition region emissions (O V and O VI) and very little gradual phase intensity for those emissions, and the east-limb flare warm corona Fe XVI emission is indicative of a quasi-periodic pulsation (QPP) flare. The west-limb flare also has large impulsive phase intensity changes for the transition region emissions plus a gradual phase component delayed after the warm coronal emissions peak. It is important to note that there is a systematic blue bias of about − 70 km/s for the corona features (Fe XVI and Si XII) for the east-limb flare, and there is a systematic red bias of about + 100 km/s for the corona features for the west-limb flare. Those biases are due to the MEGS optical wavelength shifts based on flare location. Also note in Figure 10 that the wavelength shifts of those corona features all start off more negative (more blue) by about − 150 km/s during the impulsive phase relative to flare Doppler velocity near the flare gradual phase peak. That relative change in wavelength shift is an indication of a true blue shift (outflow) for the coronal plasma, perhaps as a non-radial flow as these are limb flares. There are also wavelength shift biases for the transition-region emissions, again with a systematic negative shift for the east-limb flare and a systematic positive shift for the west-limb flare, and those large shifts are mostly due to the MEGS-B optical wavelength shift with the flares being on the limb.

**Figure 11:**
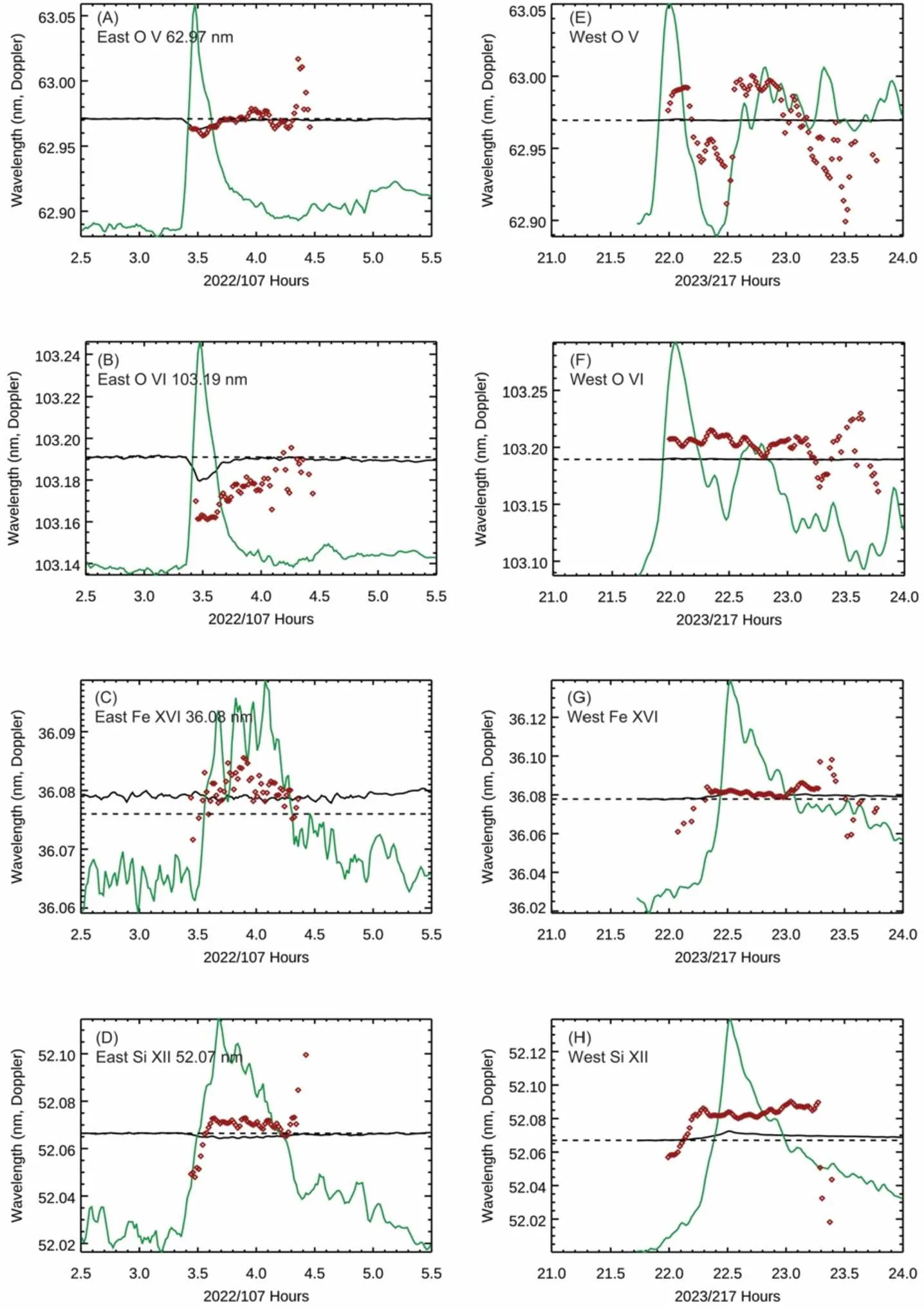
Plot of the corrected wavelength shifts for an east-limb flare (left panels) and for a west-limb flare (right panels). These are the same flares provided in Figure 10 but now includes corrections for the MEGS-B optical wavelength shifts related to flare location. The plot lines and symbols have the same definitions as those in Figure 10. The uncertainties of the corrected wavelength shifts (red diamonds) are typically between 0.0005 nm and 0.003 nm. For the worst case, their Doppler velocity uncertainties are 14.3, 8.7, 24.9, and 17.3 km/s for the emissions at 62.97, 103.19, 36.08, and 52.07 nm, respectively.

Because the EVE Level 4 Lines data processing does not have the flare location information, a correction for the MEGS optical wavelength shift needs to be made by the user of the EVE Level 4 Lines data product. To illustrate that effect of correcting the MEGS optical wavelength shift based on flare location, the data from Figure 10 are first corrected with the MEGS optical wavelength shift (Equation 2A – 2C as function of flare location and wavelength), and those corrected results are shown in Figure 11. The application of Equation 2A – 2C for the four wavelengths in Figure 10 results in larger optical wavelength shifts for the O V 62.97 nm and Si XII 52.07 nm lines and smaller shifts for the Fe XVI 36.08 nm and O VI 103.19 nm lines, as expected for the optical wavelength shifts shown in Figure 6. With the flare-location corrections made, the flare wavelength shifts (red diamonds in Figure 11) are closer to the nominal wavelengths during the gradual phase peak of the flare (coronal Si XII and Fe XVI emissions); thus, these corrected limb flares have similar blue-shift behavior during the impulsive phase as the wavelength shifts of flares near disk center. This is somewhat a surprise considering that these are limb flares and that Doppler velocities are biased along the line-of-sight; which could mean non-radial flow or very large radial flow for those limb flares. In addition, for the west-limb flare, panel E of Figure 11 for the O V emission shows an interesting trend of enhanced irradiance and a large red shift during the flare onset and impulsive phases near 22 UT and a smaller relative red shift near 22.3 UT, which is then followed by a larger red shift during the gradual phase peak near 22.5 UT. This later red shift that remains high until about 23 UT might be related to the relaxation and condensation of post-flare loops.

The relative changes in the flare Doppler velocities (wavelength shifts) relative to the velocities during the flare gradual phase peak can be effectively used to study flare dynamics regardless of whether the flare-location correction is made or not. In particular, the Fe XVI and Si XII blue shifts during the impulsive phase are about − 150 km/s relative to the gradual phase peak velocity in both Figures 10 and 11. However, if one used the wavelength shifts in Figure 10 (uncorrected for flare-location) relative to the pre-flare reference wavelength, one would falsely estimate a much larger blue shift for the east limb flare and a red shift for the west limb flare. In other words, one should make the flare-location correction for the optical wavelength shift if comparing Doppler velocities (wavelength shifts) relative to the pre-flare wavelengths. But if the flare location is not well known, then one can estimate solar flows by the difference of the wavelength shift during the impulsive phase relative to the wavelength shift at the gradual phase peak.

**Figure 12:**
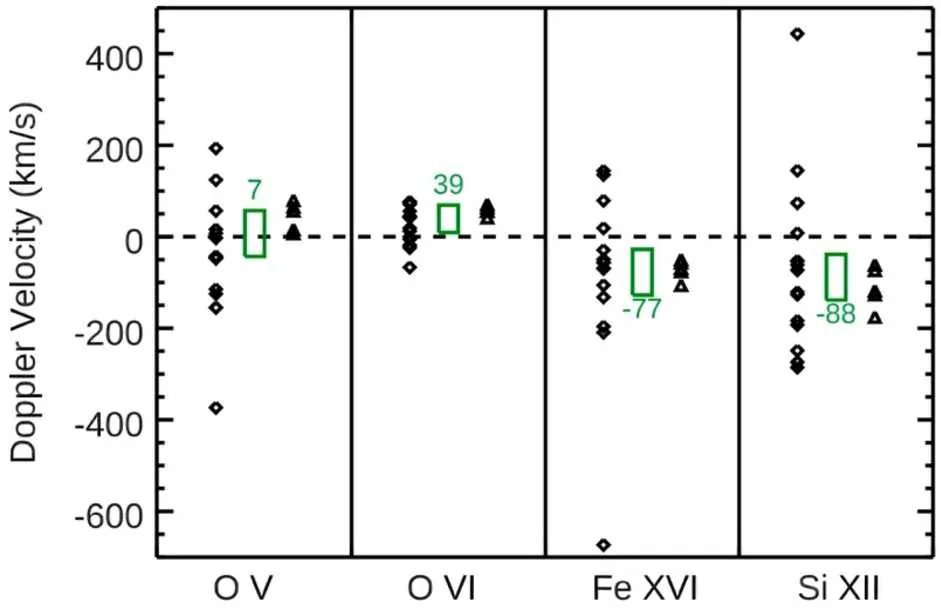
Doppler velocity results are shown for 15 X-class flares near disk center (longitude within ± 30°) as the diamonds for the O V 62.97 nm, O VI 103.19 nm, Fe XVI 36.08 nm, and Si XII 52.07 nm emission lines. There are five flares with transition region lines (O V and O VI) having positive Doppler velocities and coronal lines (Fe XVI and Si XII) having negative Doppler velocities, and those are shown as the triangles. The green box is the average of both flare sets with uncertainty indicated by the height of the box.

The EVE Level 4 Lines data product is most useful for studying the Doppler velocities from the larger flares (M and X class flares) that are often eruptive and have coronal mass ejections (CMEs) (e.g., Hock 2012; Hudson 2011; Gopalswamy et al. 2010; Priest and Forbes 2002). Although MEGS-B has observed 103 X-class flares, only 42 of them have known flare locations (Berretti et al. 2025). Of those 42 flares, 15 of them are near disk center (longitude is within ± 30°), which are more favorable to detect Doppler velocities for any radial downflows / outflows. Those 15 flare events were first corrected for the MEGS-B optical wavelength shift based on flare location and wavelength (Equation 2A – 2C), and their maximum Doppler velocities are shown in Figure 12 as the diamond symbols. The special cases for five flares when both O V and O VI transition region lines have positive Doppler velocities and when both Fe XVI and Si XII coronal lines have negative Doppler velocities are also shown in Figure 12 as the triangle symbols. The Doppler velocity averages of the means of those two sets are shown in Figure 12 as the green boxes. While the spread in the maximum Doppler velocities for those flares is positive and negative, the transition region (O V and O VI) emissions have more positive (downflow) velocities, and the coronal (Fe XVI and Si XII) emissions have more negative (upflow) velocities.

Those 15 disk-center flare Doppler velocities have no significant correlation with flare magnitude or flare location. The exception is that the flare longitude and O VI Doppler velocity have a significant correlation of 0.69 and p-value of 0.007. The MEGS-B optical wavelength shift for the O VI 103.19 nm line is essentially zero using Equation 2A – 2C (Figure 6), and this correlation with the longitude might reflect more that Equation 2A – 2C is not accurate enough for these small corrections near 100 nm. This is also a concern with Equation 2A – 2C correction for lines near 35 nm where the MEGS-B optical wavelength shift correction is also near zero. Supporting that concern, the flare longitude and Fe XVI 36.08 nm Doppler velocity have almost-significant correlation of 0.48 and p-value of 0.07. The other two lines (O V and Si XII) are not near these “zero” correction wavelengths, and their correlations with flare longitude are much smaller and their p-values are much larger (thus insignificant correlation).

## Conclusions

The EVE solar EUV spectra, despite its modest 0.1-nm spectral resolution, have proven useful to study the flare dynamics by measuring the Doppler velocities during flare events. Hudson et al. (2011) were the first to report that Doppler velocities are measurable with the EVE spectra, despite EVE not explicitly designed to measure Doppler velocities. The new EVE Level 4 Lines data product provides wavelength shift (Doppler velocity) analysis for 70 of the emission lines in the EVE spectra, and some example flare dynamics results were shown in Section 4. The eruptive flare events near solar disk center typically have red shifts (downflow) of 30 ± 20 km/s for the transition region emissions and blue shifts (upflows) of − 80 ± 50 km/s for the warm corona emissions. The flare Doppler velocities are largest during the flare’s impulsive phase and are consistent with magnetic reconnection processes described by the standard flare model (CSHKP flare model: Carmichael, Sturrock, Hirayama, Kopp, and Pneuman, e.g. Priest and Forbes 2002).

It is critical to correct the EVE Level 4 Lines data for the MEGS optical wavelength shifts that were presented in Section 3. Those corrections are a function of flare location and wavelength to obtain more accurate results for the flare Doppler velocities. The corrections for MEGS optical wavelength shifts are not as important for flares near disk center (e.g., longitude within ± 15° as shown for Figures 8 and 9) if Doppler velocity uncertainties of about 30 km/s are acceptable for one’s study. It is important to note that the MEGS optical wavelength shift corrections are not included in the EVE Level 4 Lines data product, thus a user studying flare dynamics needs to make that correction based on flare location on the solar disk. This paper only examined the larger X-class flares, so we anticipate that additional flare dynamics results will be found using this new EVE Level 4 Lines data product for the M-class and smaller flares.

## Data Availability

The primary data shown in this manuscript are SDO EVE solar EUV irradiance measurements that are available as public EVE data products (version 8.1) at http://lasp.colorado.edu/eve/. The GOES XRS data are from https://www.ncei.noaa.gov/products/goes-r-extreme-ultraviolet-xray-irradiance/, and SDO AIA solar EUV images are from http://jsoc.stanford.edu/AIA/AIA_jsoc.html and also https://suntoday.lmsal.com/suntoday/.
